# Supplementary material for: Therapeutic Anti-Tumor Efficacy of DC-Based Vaccines Targeting TME-Associated Antigens Is Improved When Combined with a Chemokine-Modulating Regimen and/or Anti-PD-L1
Source: Vaccines (Basel). 2024 Jul 15;12(7):777. doi: 10.3390/vaccines12070777 (PMC11281486; doi:10.3390/vaccines12070777)
Supplement: Supplementary file 1 [file vaccines-12-00777-s001.zip › vaccines-3045037-supplementary.pdf]

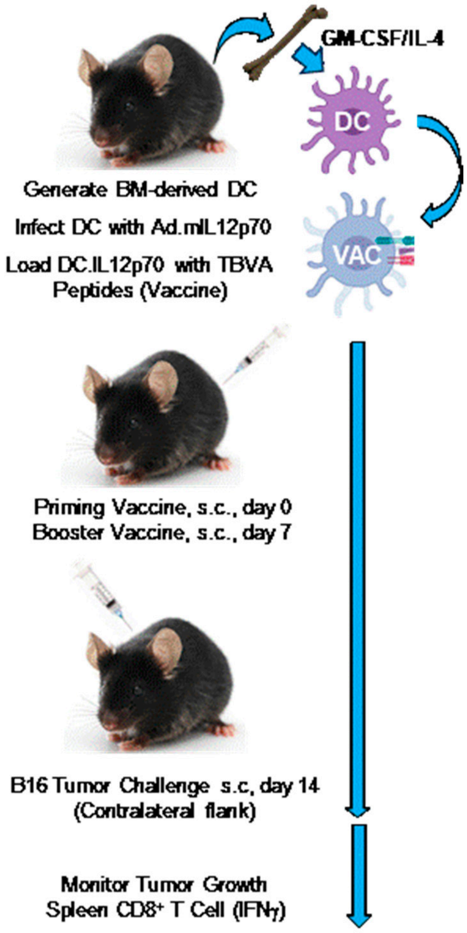
GM-CSF/IL-4
DC
VAC
Generate BM-derived DC
Infect DC with Ad.mIL12p70
Load DC.IL12p70 with TBVA Peptides (Vaccine)
Priming Vaccine, s.c., day 0
Booster Vaccine, s.c., day 7
B16 Tumor Challenge s.c, day 14
(Contralateral flank)
Monitor Tumor Growth
Spleen CD8+ T Cell (IFNγ)

A.

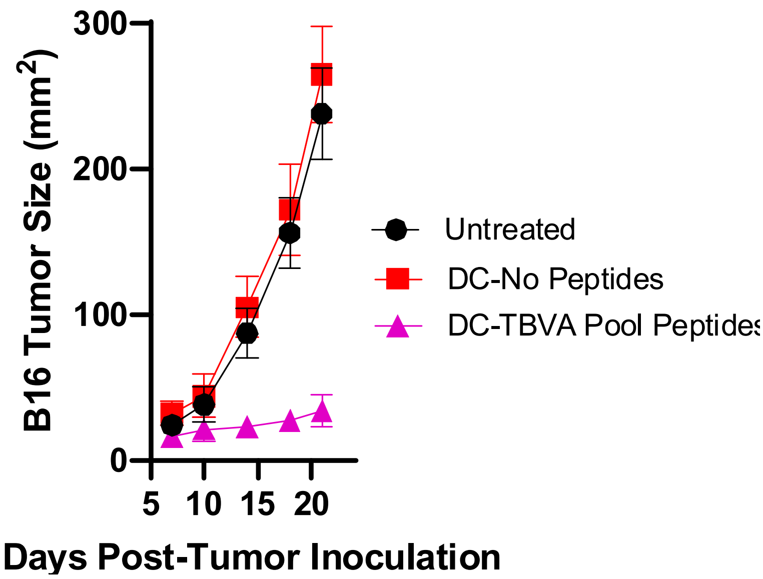
B16 Tumor Size (mm2)
300
200
100
0
5
10
15
20
Days Post-Tumor Inoculation
Untreated
DC-No Peptides
DC-TBVA Pool Peptides

B.

TBVA Peptide	AA Sequence	H-2^b Restrict.*	IFN-g (pg/ml)**	Reference
mDLK1 (262-270)	SGYGLTYRL	K^b	338 $\pm$ 51	This report
mEphA2 (671-679)	FSHHNIIRL	D^b	495 $\pm$ 42	Ref. 25
mHBB (34-42)	VVYPWTQRY	K^b	118 $\pm$ 21	This report
mNRP1 (864-871)	MSALGVLL	K^b	271 $\pm$ 53	This report
mRGS5 (71-78)	NSYGFASF	K^b	94 $\pm$ 23	This report
mTEM1 (76-85)	VGPANGLLWI	D^b	253 $\pm$ 41	This report
E7 (49-57)	RAHYNIVTF	D^b	14 $\pm$ 17	Ref. 26

C.

Figure S1. Screening TBVA-derived peptides for the Immunogenicity/Prophylactic Anti-Tumor Efficacy of DC12/Pool Peptide Vaccines. (**A**) C57BL/6 mice (5/group) received priming/booster vaccination with PBS, 10^6 syngenic DC.IL12 (no peptide) cells or 10^5 syngenic DC.IL12/TBVA pool peptide cells s.c. on the left flank on days 0 and 7. On day 14, B16 tumor cells (10^5) were injected s.c. on the right flank. In (**B**), tumor growth was then monitored every 3-4 days, with mean tumor area (+/-SD) reported in mm^2; $p < 0.05$ for DC.IL12/TBVA pool peptide vs. both control cohorts. On day 21 post-tumor inoculation, animals were euthanized and splenocytes harvested, with individual peptides then used to restimulate splenic T cells in vitro for 48h as outlined in Materials and Methods. Cell free supernatants were then evaluated for IFNγ content using a commercial ELISA (reported in pg/ml). *Preferred MHC class I allele presenting the indicated peptide based on algorithm assessment (https://www.iedb.org/); ** $p < 0.05$ for each TBVA peptide vs. irrelevant control HPV-16 E7 peptide.

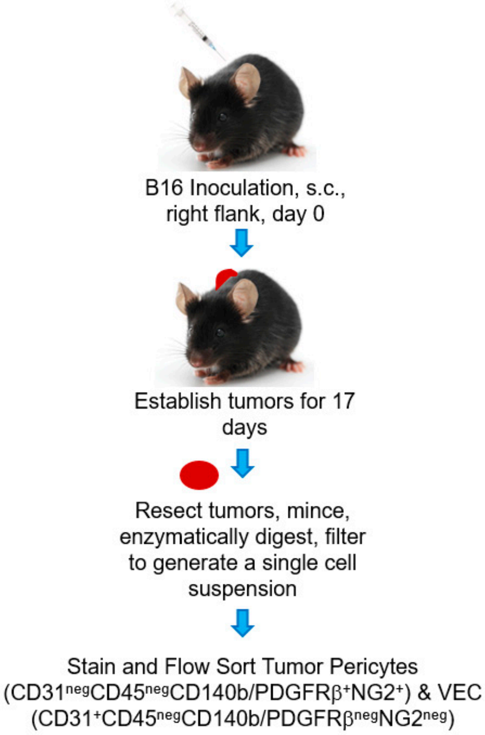
B16 Inoculation, s.c., right flank, day 0
Establish tumors for 17 days
Resect tumors, mince, enzymatically digest, filter to generate a single cell suspension
Stain and Flow Sort Tumor Pericytes (CD31negCD45negCD140b/PDGFRβ+NG2+) & VEC (CD31+CD45negCD140b/PDGFRβnegNG2neg)

A.

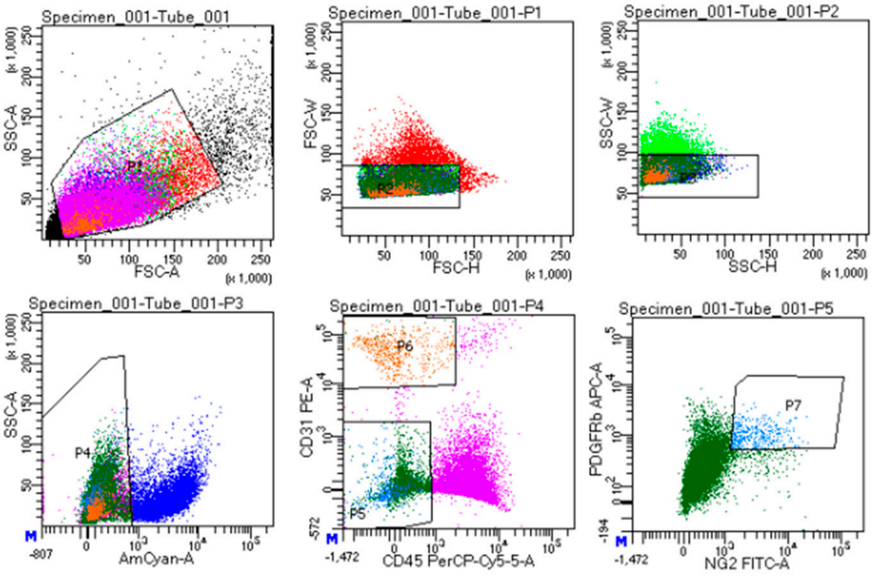
Specimen_001-Tube_001
SSC-A
FSC-A
P1
Specimen_001-Tube_001-P1
FSC-W
FSC-H
P2
Specimen_001-Tube_001-P2
SSC-W
SSC-H
Specimen_001-Tube_001-P3
SSC-A
AmCyan-A
P4
Specimen_001-Tube_001-P4
CD31 PE-A
CD45 PerCP-Cy5-5-A
P6
P5
Specimen_001-Tube_001-P5
PDGFRb APC-A
NG2 FITC-A
P7

B.

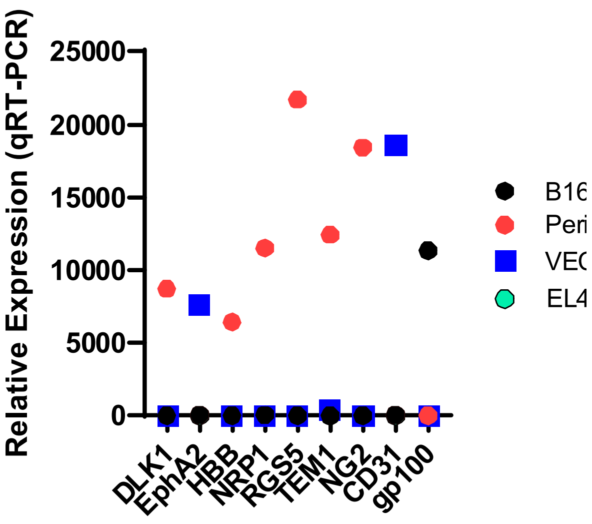

C.

Figure S2. Flow sorting of B16 Tumor-Associated Pericytes and VEC. (A) C57BL/6 mice (n = 5) were injected s.c. on the right flank with B16 melanoma cells (10^5) on day 0 and tumors allowed to establish and grow. On day 17, animals were euthanized, and tumors harvested. Tumors were minced, pooled and then enzymatically digested as described in Materials and Methods. Single cell suspensions were stained with fluorescently-conjugated antibodies against mNG2, mCD31, mCD45 and mCD140b/PDGFRβ (see Materials and Methods for details) prior to flow sorting. (B) Gating strategy for isolation of pericytes (CD31^{neg}CD45^{neg}CD140b/PDGFRb^{+}NG2^{+}) and VEC (CD31^{+}CD45^{neg}CD140b/PDGFRβ^{neg} NG2^{neg}). (C) Relative transcript expression for the indicated target gene products in cultured B16 melanoma cells, cultured EL4 thymoma cells and flow sorted tumor-associated pericytes and VEC (qRT-PCR). qRT-PCR primers are listed in Table S1.

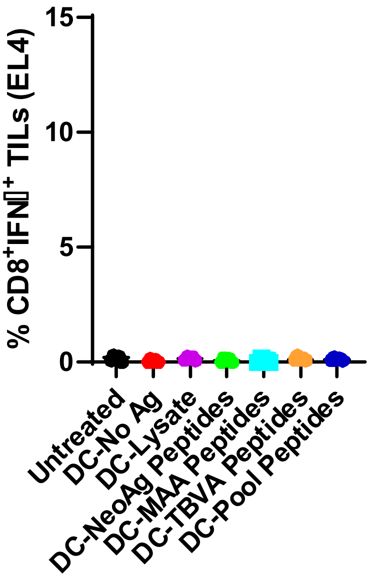

Figure S3. B16 TIL harvested from untreated mice, or mice treated with various DC-based vaccines fail to demonstrate IFNγ (ICS) responses against the irrelevant syngenic EL4 thymoma target cell line. Experiments were performed as outlined in Fig. 1 and Materials and Methods. NS for all groups vs. untreated.

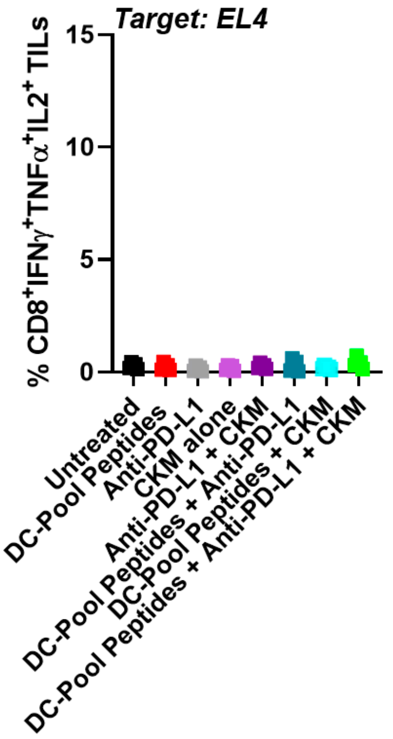

Figure S4. B16 TIL harvested from untreated mice, or mice treated with DC-Pool Peptide vaccines +/- CKM +/- anti-PD-L1 fail to exhibit polyfunctional responses against the irrelevant syngenic EL4 thymoma target cell line. Experiments were performed as outlined in Fig. 4 and Materials and Methods. NS for all groups vs. untreated.

Table S1. qRT-PCR primers used in this study.

Target Transcript	Forward Primer	Reverse Primer
mDLK1	TGTGACCCCCAGTATGGATT	CCAGGGGCAGTTACACACTT
mEphA2	TATGGCAAAGGGTGGGACCT	TACACCCAGTTGGTGCGGA
mHBB	TGAATCACTTGGACAGCCTCAA	CATATTGCCCAGGAGCCTGAAG
mNRP1	GACAAATGTGGCGGGACCATA	TGGATTAGCCATTCACACTTCTC
mRGS5	ACAAGCCCTCACTGGAGGA	CTTTTGAAGCTGGCAAATCC
mTEM1/CD248	AGGCCCCTATGTCTCCAGAT	GGGTGTTGATGGGCTTTAGA
mNG2	AAACACAGGCCTGCAAATCT	GCTCGATGGTGTAGACCAAGT
mCD31	GGAACGAGAGCCACAGAGAC	TGCACTGCCTTGACTGTCTT
mgp100	CTCTTGTTTCCTGTGGTTCCT	GTAGTGGTTCCTTGCCTAGATG
